# Epigallocatechin gallate (EGCG) attenuates myocardial hypertrophy and fibrosis induced by transverse aortic constriction via inhibiting the Akt/mTOR pathway

**DOI:** 10.1080/13880209.2021.1972124

**Published:** 2021-10-04

**Authors:** Yue Cui, Yongqiang Wang, Gang Liu

**Affiliations:** a Department of Medicine, Tianjin HuanHu Hospital, Tianjin, China; b Intensive Care Unit, Tianjin First Central Hospital, Tianjin, China

**Keywords:** Cardiac hypertrophy, Akt/mTOR, epigallocatechin gallate (EGCG)

## Abstract

**Context:**

Epigallocatechin gallate (EGCG) is the most abundant catechin from tea. Previous studies have indicated EGCG has a cardioprotective effect.

**Objective:**

This manuscript mainly explores the role of EGCG in pressure-overload cardiac hypertrophy and its mechanism related to the Akt/mTOR pathway.

**Methods and methods:**

Transverse aortic constriction (TAC) was utilized to establish the cardiac hypertrophy mice model. C57BL/6 mice were assigned into 6 groups. Starting from the first day after surgery, mice received different doses of EGCG (20, 40, 80 mg/kg) or vehicle orally for four weeks. Heart weight to body weight (HW/BW) ratio and heart weight to tibia length (HW/TL) ratio as well as hematoxylin-eosin staining were utilized to evaluate cardiac hypertrophy. Masson’s trichrome and Sirius red staining were used to depict cardiac fibrosis. The expressions of fibrosis and hypertrophy-related markers and Akt/mTOR pathway were quantified by western blot and qRT-PCR.

**Results:**

EGCG significantly attenuated cardiac function shown by decreased HW/BW (TAC, 6.82 ± 0.44 vs. 20 mg/kg EGCG, 5.53 ± 0.45; 40 mg/kg EGCG, 4.79 ± 0.32; 80 mg/kg EGCG, 4.81 ± 0.38) and HW/TL (TAC, 11.94 ± 0.69 vs. 20 mg/kg EGCG, 11.44 ± 0.49; 40 mg/kg EGCG, 8.83 ± 0.58; 80 mg/kg EGCG, 8.98 ± 0.63) ratios as well as alleviated cardiac histology. After treatment, hemodynamics was improved, cardiac fibrosis was attenuated. The activated Akt/mTOR pathway was inhibited by EGCG.

**Discussion and conclusions:**

EGCG plays a protective role in the TAC model by regulating the Akt/mTOR pathway, which provides a theoretical basis for its clinical treatment.

## Introduction

Heart failure is a major cause of death all around the world; it has been determined that maladaptive remodeling of the heart, which occurs after hemodynamic loads, is the element responsible for heart failures, such as those after myocardial infarction or chronic hypertension. Myocardial hypertrophy is thought to be an adaptive response to pressure or volume stress and many other problems. Initially, it is adaptively against increased workload or defects in the efficiency of contractile machinery. However, in the longer term, hypertrophy growth may result in the development of heart failure and sudden death (Frey et al. 2004). Myocardial hypertrophy is also defined as the increase in ventricular myocardial mass and is a common disease that can be found in the majority of patients with heart failure with preserved ejection fraction (HFpEF, Heinzel et al. 2015). Myocardial hypertrophy is often characterised by elevated fibrosis, mostly reactive interstitial fibrosis. The increases in total collagen expression and cross-linking were associated with diastolic dysfunction, and the fibrosis may result in the progression of left ventricular hypertrophy to heart failure (Falcão-Pires et al. 2011). Fibrillar collagens are the most abundant collagen types in the heart, among which type I/III accounting together for over 90% of the total collagen. These two types of collagen functions differ from each other. Type I collagen molecules assemble into thick fibers while type III forms a fine fibrils network (Weber et al. 1994). Connective tissue growth factor (CTGF) has been indicated to regulate many signalling pathways and contribute to tissue remodeling and fibrosis, and the inhibition of CTGF expression can reverse fibrosis after significant collagen deposition (Lipson Kenneth et al. 2012). CTGF has been reported to interact with other molecules to positively or negatively alter the signal transduction pathways in with they are involved, and when connected with fibronectin, CTGF promotes fibroblast (Chen et al. 2004).

Recent research is dedicated to the investigation of downstream effector pathways involved in the progression of cardiac hypertrophy and heart failure, Akt and mammalian target of rapamycin (mTOR) are included (Aoyagi and Matsui 2011). A previous study indicated that Akt/mTOR axis plays an important role in eccentric hypertrophy during volume overload in response to diastolic wall stress. The study also pointed that mTOR activity regulates the rate of eccentric hypertrophy progression (Ikeda et al. 2015). Acute Akt activation itself may protect cardiomyocytes from apoptosis *in vitro* and *in vivo*, however, chronic Akt overexpression is sufficient to contribute to myocardial hypertrophy in transgenic mice with preserved systolic function and cardioprotection to massive cardiac dilatation and sudden death. The mTOR is one of the downstream targets of Akt in hypertrophic signalling, mTOR-induced acceleration of protein translation can enhance cell growth and mass. The mTOR activation further activates key protein translation regulators such as p70S6 kinase and 4EBP1/eIF4E, thereby enhancing protein synthesis, a classic feature of cardiomyocyte hypertrophy (Frey et al. 2004).

Catechins are a category of bioactive chemicals rich in tea. Tea catechins and polyphenols are capable of scavenging effects for reactive species, which have recently acquired multiple attention for the treatment of cardiovascular attention (Li H-L et al. 2006). Epigallocatechin gallate (EGCG) is the most abundant among catechins (Higdon and Frei 2003; Negri et al. 2018). A previous study indicated that EGCG alleviated pressure overload-induced cardiac hypertrophy by inhibiting cardiomyocytes apoptosis and oxidative stress, possibly through inhibition of p53 induction and Bcl-2 decrease (Sheng et al. 2007). Research also revealed that EGCG exhibited a cardioprotective effect by inducing NO production, inhibited the proliferation of cardiac fibroblasts both *in vitro* and *in vivo*, thereby preventing myocardial fibrosis in cardiac hypertrophy (Sheng et al. 2006). However, the study for the underline mechanism of EGCG associated with AKT/mTOR signalling in cardiac hypertrophy and fibrosis is still unknown.

Therefore, elucidating the relationship between EGCG and underlying signalling connections is important for the development of a potential therapeutic strategy for cardiac hypertrophy. Several signal pathways have been reported to be involved in the hypertrophic responses in cardiomyocytes (Sugden 1999; Molkentin and Dorn II 2001; Frey and Olson 2003). This study will focus on the role of EGCG in the development of myocardial hypertrophy and its treatment effect associated with myocardial fibrosis, as well as its interaction with the Akt/mTOR signal pathway that plays a pivotal role in cardiac hypertrophy and regulating mRNA translation and cell growth. Efforts are made to provide a more comprehensive understanding of EGCG treatment for cardiac hypertrophy.

## Material and methods

### Animals

Male C57BL/6 mice weighing between 23–27 g and aged 8–10 weeks were purchased from Beijing HFK bioscience (Beijing, China). The animal treatment and experiment procedures conducted in this study were all approved by an ethical committee of local and strictly following the guidelines of the Institutional Animal Ethics Committee (IAEC). Mice were housed for accommodation for one week before surgery. During accommodation, the environment was under control with a temperature of around 22 °C environments with 12/12 light and dark cycles. Mice were fed with normal chow and allowed free access to water. After accommodation, mice were assigned into 6 groups (control, sham, TAC, TAC + EGCG 20 mg/kg, TAC + EGCG 40 mg/kg, TAC + EGCG 80 mg/kg) with 20 mice for each group (Yang et al. 2014). Transverse aortic constriction (TAC) was carried out according to a previous study (Tagashira et al. 2010). Briefly, mice were anaesthetized with pentobarbital sodium (0.077 g/kg). The mice were equipped with an endotracheal tube connected to a rodent ventilator. The chest cavity was opened and then the aortic arch was isolated, the transverse aorta was isolated and constricted by a 7-0 silk suture ligature against a 27-gauge needle. The needle was then withdrawn immediately to make an aortic constriction with 0.4 mm diameter. After the surgery, each mouse chest was closed. Control group mice experienced none of the experiment and sham-operated animals underwent the same procedure with the omission of aorta ligation. Starting from the first day after surgery, mice received different doses of EGCG (20, 40, 80 mg/kg) or vehicle orally for four weeks before the therapeutic evaluation for myocardial hypertrophy. EGCG was purchased from Aladdin Reagent Co., Ltd. (Shanghai, China).

### Measurement of cardiac hypertrophy

At the end of 4 weeks of EGCG or vehicle administration, mice were weighed and then sacrificed. The thoracic cavity was opened followed by the harvest of the heart. The heart was also weighed and compared with the body weight, the cardiac hypertrophy was estimated as HW-BW ratio (mg/g), and HW-TL (mg/mm).

### Serum biochemical analysis

Blood samples were left to clot at room temperature and centrifuged at 3,000 rpm for 15 min. The serum was separated and stored at −20 °C for further biochemical analysis. CK-MB, cTnT, and cTnI were evaluated according to the protocol using the kits (Roche Diagnostics).

### Hemodynamic detection

As described previously (Liu et al. 2013), the changes in cardiac function were assessed by observing hemodynamics. After mice were anaesthetized by isoflurane inhalation, the right carotid artery was visualized the intubated with a Midro-Tip catheter (Millar) which was connected to a pressure sensor. The catheter was inserted into the left ventricle which was confirmed by the alteration of the pressure curve plotted by the Powerlab 4/25 Biological Analysis system. The left ventricular systolic pressure (LVSP) and left ventricular end-diastolic pressure (LVEDP), mean arterial pressure (MAP), the maximum rate of left ventricular pressure decay (−dp/dt), and mean flow velocity (V_mean_) were measured and recorded.

### Histology staining

In order to measure cardiomyocyte area and cardiac fibrosis, heart tissues were separated and washed with ice-cold PBS. The tissues were then fixed in 4% paraformaldehyde and embedded in paraffin and then sliced into serial sections (3 μm). The procedures of hematoxylin and eosin (H&E), Masson’s trichrome (Sigma, USA) and Sirius red (Polysciences, USA) staining were following the previous studies and manufacturer’s instructions (Chen et al. 2011). A microscope was used to observe the pathological changes.

### Western blot analysis

Heart tissues were dissected and rapidly frozen in liquid nitrogen and stored at −80 °C before use. For an experiment, tissue lysates were prepared by the method previously described (Tagashira et al. 2010). An equal amount of protein (25 μg) was subjected to SDS-PAGE and transferred onto PVDF membranes (Millipore). Membranes were blocked with 5% low-fat milk in TBST and then incubated with primary antibodies overnight at 4 °C. The antibodies were all purchased from Proteintech (Wuhan, China) without specific notification: ANP (Cat. No. 10843-1-AP), BNP (Cat. No. 13299-1-AP), a-MHC (Abcam, ab134189), β-MHC (Abcam, ab23990), Collagen I (Cat. No. 14695-1-AP), Collagen III (Cat. No. 22734-1-AP), fibronectin (Cat. No. 15613-1-AP), CTGF (Cat. No. 23936-1-AP), GAPDH (Abcam, ab181602), p-ATK (Cat. No. 66444-1-Ig), AKT (Cat. No. 10176-2-AP), p-mTOR (Abcam, ab109268)), mTOR (Cat. No. 20657-1-AP), p-ERK (Cell Signalling Technology, Cat. No. 9101), ERK (Cat. No. 16443-1-AP). After incubation with primary antibodies, the membrane was then exposed to anti-rabbit or anti-mouse horseradish peroxidase-conjugated secondary antibodies for 2 h at 25 °C, and blots were visualized using ECL immunoblotting detecting system.

### Quantitative real-time PCR analysis

Primers were designed according to the ANP, BNP, α-MHC, β-MHC, collagen I, collagen III, fibronectin, and CTGF sequences reported in GeneBank and synthesized by General Biosystems (Anhui, China). Primers used in PCR are listed in Table 1. Total RNA from heart tissue was isolated with Trizol reagent (Invitrogen). Reverse transcription was performed with 2 µg of RNA using the Superscript II RNase H_Reverse Transcriptase (TAKARA, Beijing, China). First-strand DNA was synthesized by reverse transcription. QRT-PCR analysis was performed following the real-time fluorescence quantitative PCR kit (TAKARA, Beijing, China). The experiment protocols were strictly following the manufacturer’s instructions.

**Table 1.** Primer sequences used in qRT-PCR experiment.

Gene	Forward	Reverse
*ANP*	5′-GAGAAGATGCCGGTAGAAGA-3′	5′-AAGCACTGCCGTCTCTCAGA-3′
*BNP*	5′-CTGCTGGAGCTGATAAGAGA-3′	5′-TGCCCAAAGCAGCTTGAGAT-3′
*β-MHC*	5′-GATGCTGTCCGTGCCAATGACGA-3′	5′-TGATGAGGCTGGTGTTCTGCGAGT-3′
*a-MHC*	5′-CTCTGCTCTGTGACAGGCTTCTACC-3′	5′-CACCAAGCTCTGGGATCATTTCC-3′
*collagen I*	5′-CAGACCCAAGGACTATGAAGTTGATGC-3′	5′-AATCCAGTAGTAATCGCTGTTCCACTCT-3′
*collagen III*	5′-GGTTTGGAGAATCTATGAATGGTGGTT-3′	5′-CAGTGGTATGTAATGTTCTGGGAGGC-3′
*fibronectin*	5′-GAGGCACAAGGTTCGGGAAGAGG-3′	5′-TGGCGTAATGGGAAACCGTGTAA-3′
*CTGF*	5′-CTGTGCCTGCCATTACAACTGTCC-3′	5′-TCGTGTCCCTTACTTCCTGGCTTTAC-3′
*GAPDH*	5′-CTTCAACAGCAACTCCCATTCTTCC-3′	5′-GGGTGGTCCAGGGTTTCTTACTCC-3′

### Statistical analysis

All the experimental results were expressed as means ± SD. Differences among groups were tested by one-way ANOVA. Comparisons between the two groups were performed by unpaired Student’s *t*-test. ANOVA with *post hoc* Fisher’s protected least significant difference test was used for comparison among groups. A value of *p* < 0.05 was considered to be significantly different.

## Results

### EGCG reversed cardiac hypertrophy in mice with TAC

**Figure 1.**
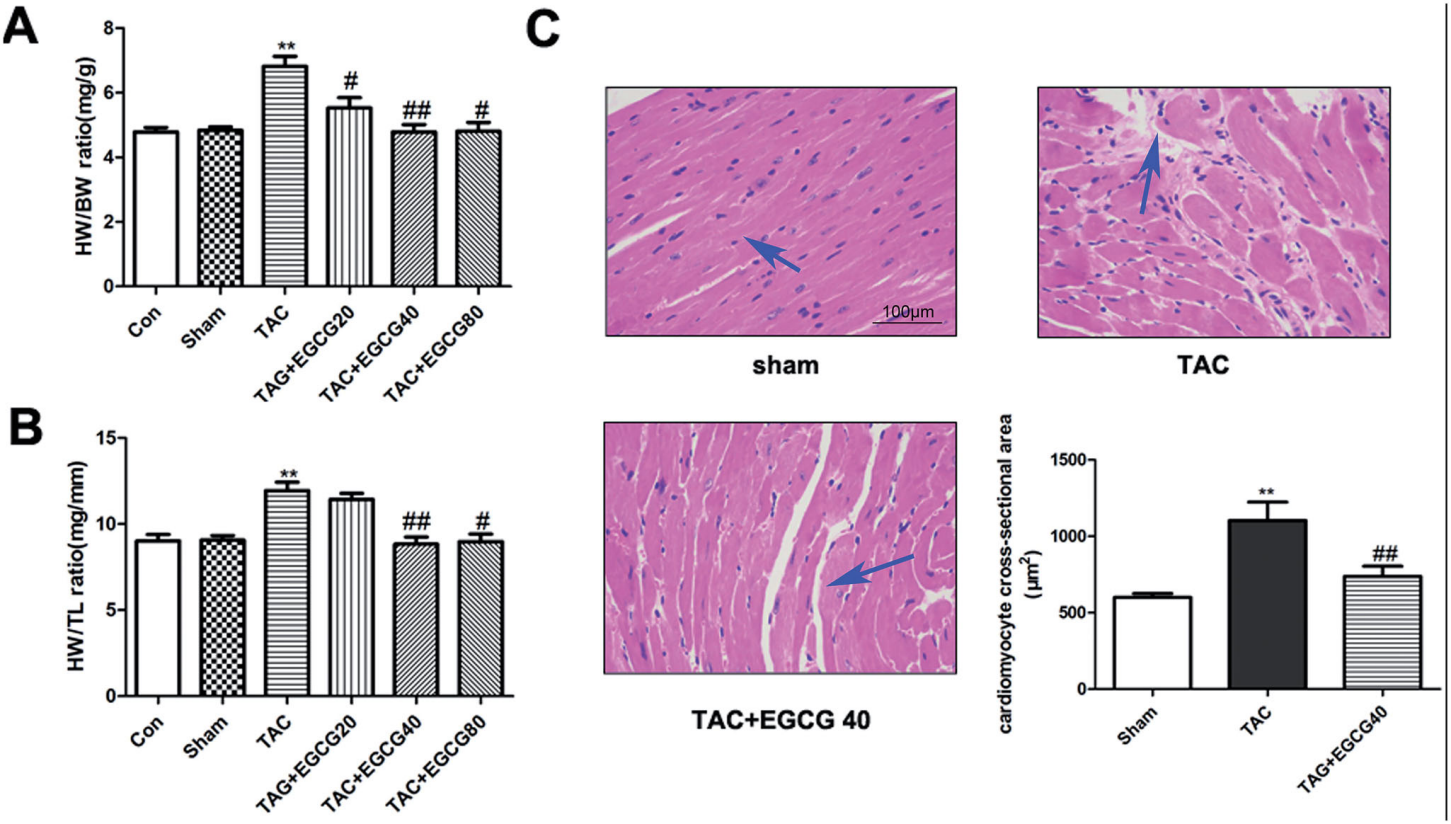
EGCG reverses cardiac hypertrophy in mice with TAC. (A) Heart weight to body weight (HW/BW) ratio. (B) Heart weight to tibia length (HW/TL) ratio. (C) H&E staining of different groups. **p < 0.01 as compared with the sham group. ^#^*p* < 0.05, ^##^*p* < 0.01 as compared with the TAC group.

To determine whether EGCG alleviates cardiac hypertrophy under pressure overload, mice were administrated with EGCG for 4 weeks after TAC surgery. According to the results (Figure 1(A,B)), TAC mice treated with vehicle control developed cardiac hypertrophy which exhibited elevated HW/BW and HW/TL ratios (*p* < 0.001). EGCG treatment significantly restored both elevated ratios (*p* < 0.01). In the results above, the effect of 40 and 80 mg/kg EGCG dosages exhibited no significant differences but better than the 20 mg/kg group on treatment for cardiac hypertrophy. Therefore, we chose 40 mg/kg as the experimental dose in the following test. The cardioprotective effect of EGCG against cardiac hypertrophy was further confirmed by morphological analysis. The result of H&E staining (Figure 1(C)) indicated that EGCG (40 mg/kg) decreased myocyte cross-sectional area which was increased by TAC pressure overload surgery (*p* < 0.001).

### EGCG improved the cardiac function in mice with TAC

**Figure 2.**
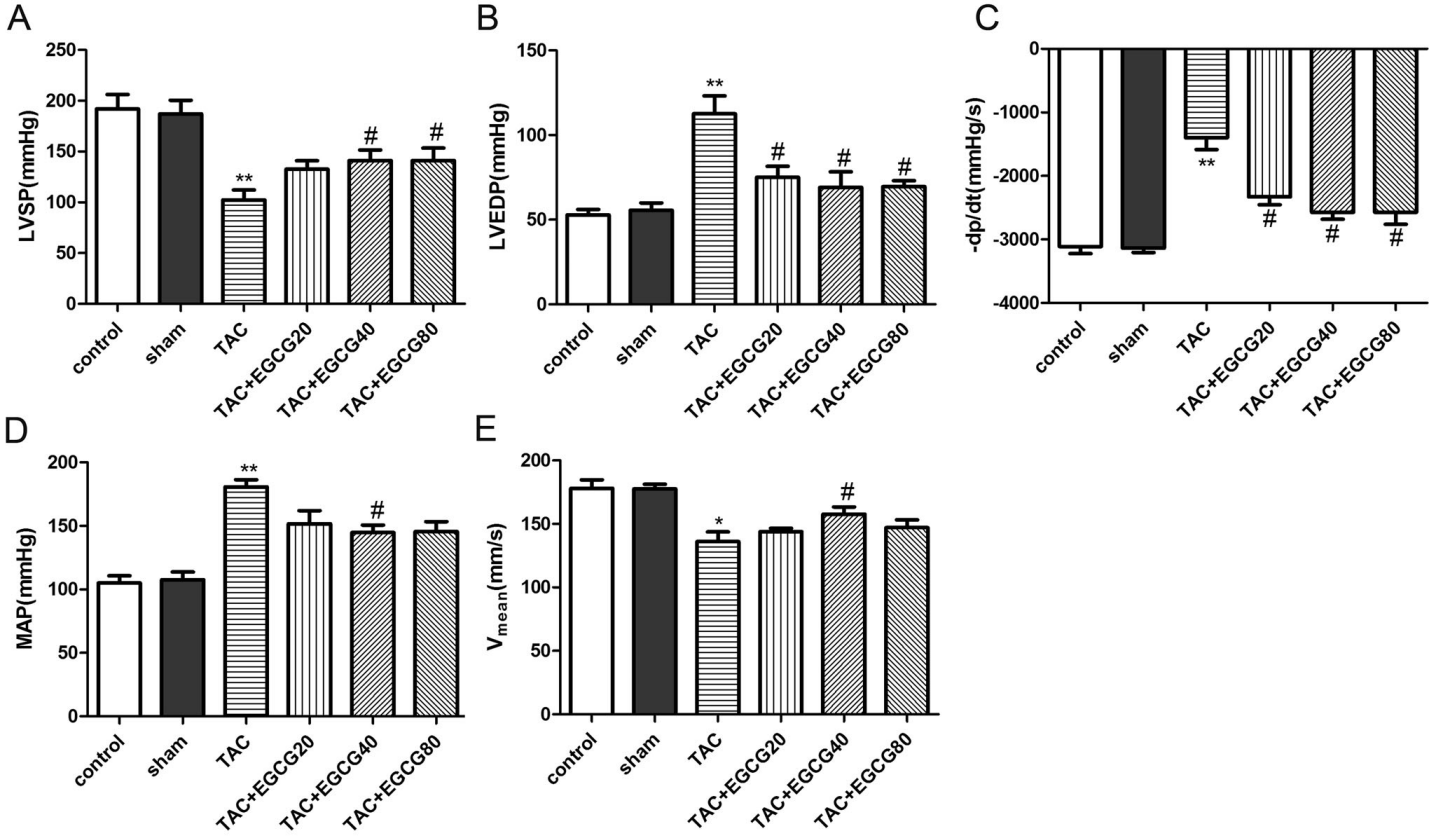
EGCG improves cardiac function in mice with TAC. Hemodynamic parameters (LVSP (A), LVEDP (B), -dp/dt (C), MAP (D), V_mean_ (E)) of mice in different groups were detected, respectively. The levels of CK-MB (F), cTnT (G), and cTnI (H) in the serum were detected by kits. *p < 0.05, **p < 0.01, as compared with the sham group. ^#^*p* < 0.05 as compared with the TAC group.

As shown in Figure 2, the markedly increased MAP and impaired cardiac functions confirmed the successful establishment of the TAC model. Compared with the sham group, the LVSP and V_mean_ decreased significantly while the LVEDP, MAP, and –dp/dt increased in mice subjected to TAC. However, the values of LVSP and V_mean_ were dramatically increased while LVEDP as well as –dP/dt were significantly reduced in EGCG (40 mg/kg) administrated mice exposed to TAC. Taken together, we found that EGCG (40 mg/kg) could improve cardiac function in TAC mice (*p* < 0.05). Then, serum markers of heart failure were detected by kits. As shown in Figure 2, compared with the sham group, the levels of CK-MB, cTnT, and cTnI in the TAC group were increased significantly. EGCG group could significantly decrease the levels of CK-MB, cTnT, and cTnI compared with the TAC group.

### EGCG reduced expression of hypertrophic marker genes in mice with TAC

**Figure 3.**
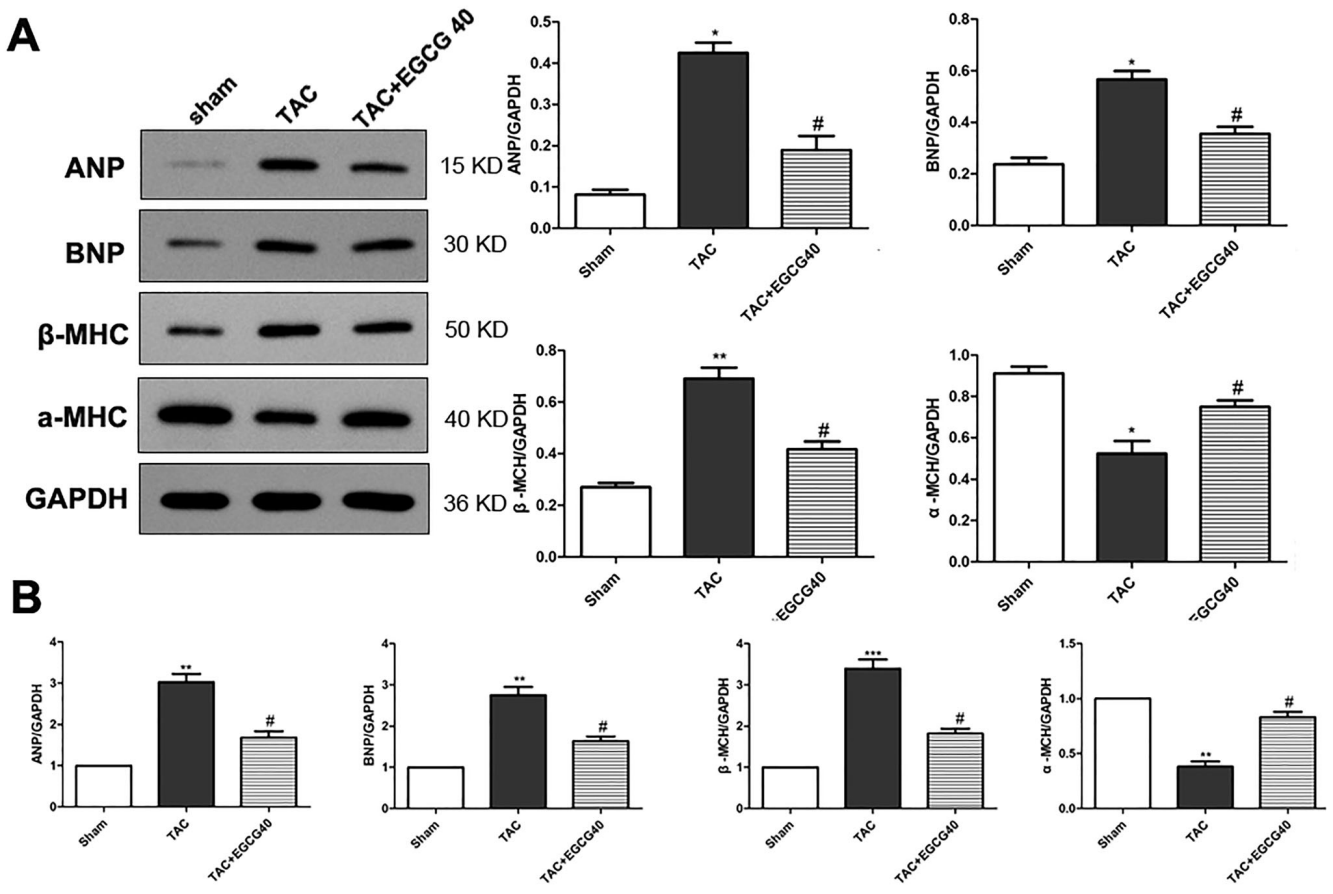
EGCG reduces the expression of hypertrophic marker genes in mice with TAC. (A) Western blot assay for protein expression of hypertrophic markers. (B) QRT-PCR assay for mRNA expressions of hypertrophic markers. *p < 0.05, **p < 0.01, ***p < 0.001 as compared with the sham group. ^#^*p* < 0.05 as compared with the TAC group.

In the present experiment, the protein expression levels of hypertrophic markers, including atrial natriuretic peptides (ANP), B-type natriuretic peptides (BNP), and β-myosin heavy chain (MHC), were increased by TAC surgery in TAC group mice (*p* < 0.001) (Figure 3(A)). However, the level of α-MHC was decreased by the stimulation of pressure overload. Treatment with EGCG (40 mg/kg) reversed the protein expression levels with decreased hypertrophic markers and increased α-MHC expression level (*p* < 0.01). The mRNA expression levels detected by qRT-PCR further verified the hypertrophic markers expressions and indicated that EGCG attenuated cardiac hypertrophy.

### EGCG reduced fibrotic and condensed collagen-deposition areas in mice with TAC

**Figure 4.**
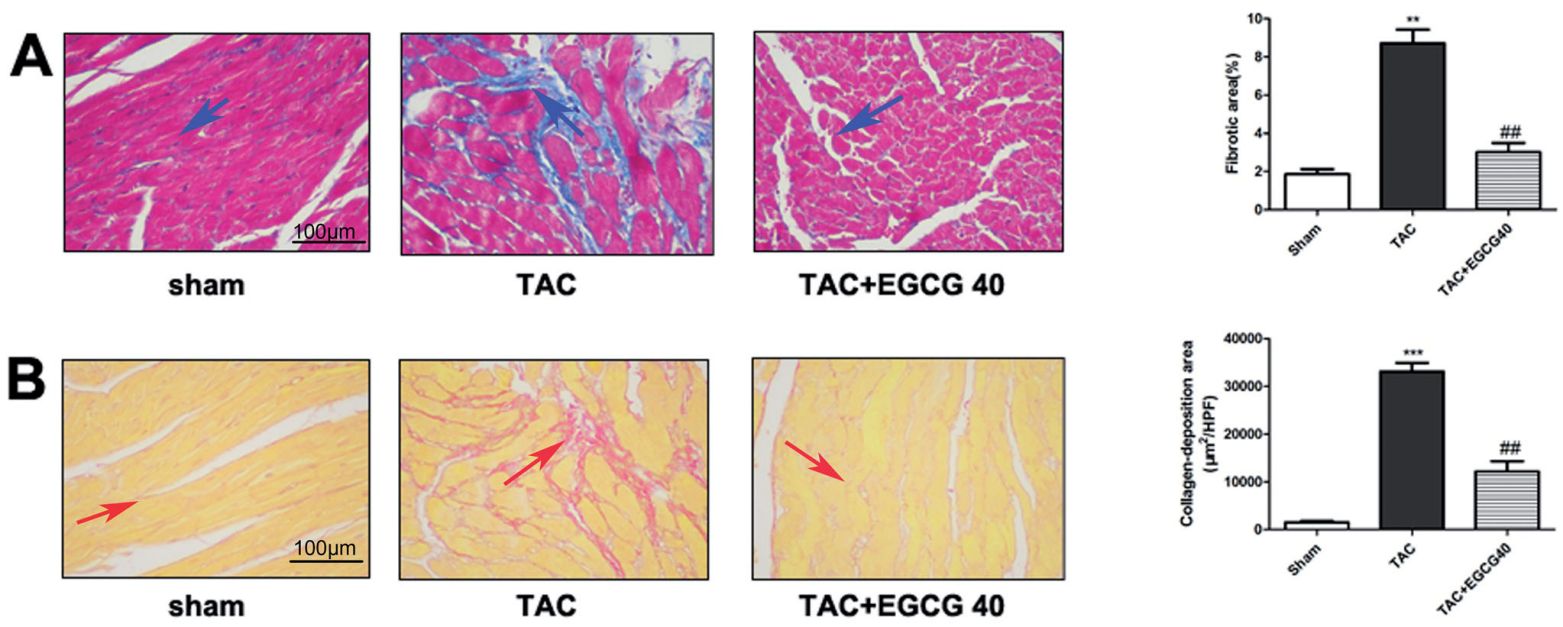
EGCG reduces fibrotic and condensed collagen-deposition areas in mice with TAC. (A) Masson’s trichrome staining of different groups. (B) Sirius red staining of different groups. ***p < 0.001 as compared with the sham group. ^##^*p* < 0.01 as compared with the TAC group.

To determine whether EGCG attenuates fibrosis and collagen deposition following TAC, Masson’s trichome staining and Sirius red staining were performed. As the results are shown in Figure 4(A), a larger degree of cardiac interstitial and perivascular fibrosis was exhibited in the TAC group than that of normal and sham control groups. The administration of EGCG (40 mg/kg) significantly decreased cardiac fibrosis compared with vehicle control group mice (*p* < 0.001). Sirius red staining results represent collagen disposition situations in heart tissues. A similar result was observed in Sirius red staining that EGCG reduced the collagen deposition and fibrosis caused by TAC (*p* < 0.001) (Figure 4(B)).

### Epigallocatechin gallate (EGCG) attenuated expression of cardiac fibrosis marker genes in mice with TAC

**Figure 5.**
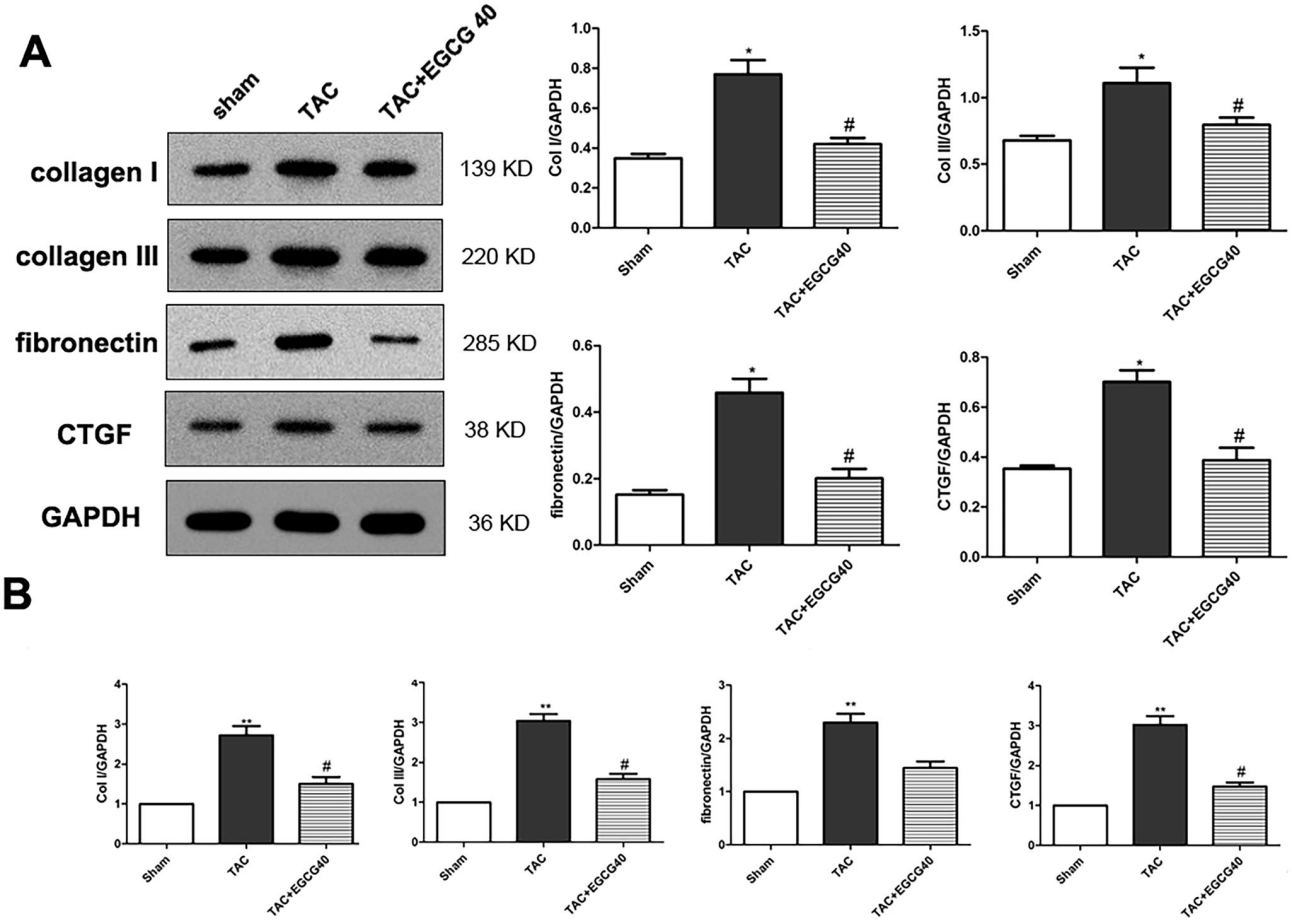
EGCG attenuates expression of cardiac fibrosis marker genes in mice with TAC. Western blot assay for protein expression of cardiac fibrosis markers. (B) QRT-PCR assay for mRNA expression of cardiac fibrosis markers. *p < 0.05, **p < 0.01 as compared with the sham group. ^#^*p* < 0.05 as compared with the TAC group.

Cardiac fibrosis is recognised as an important marker for hypertrophic pathology. In our present study, we further investigated the protein and mRNA expression levels of fibrosis markers. Here, we found that TAC surgery triggered the protein overexpression of collagen I, collagen III, fibronectin, and CTGF (Figure 5(A)). Nevertheless, EGCG (40 mg/kg) administration decreased these protein expressions, indicating the attenuated fibrosis in cardiac hypertrophic mice (*p* < 0.01). The qRT-PCR results demonstrated the similar mRNA expressions of these genes and further verified the results in western blot (Figure 5(B)).

### EGCG attenuates the activation of AKT/mTOR signalling and ERK1/2 phosphorylation in mice with TAC

**Figure 6.**
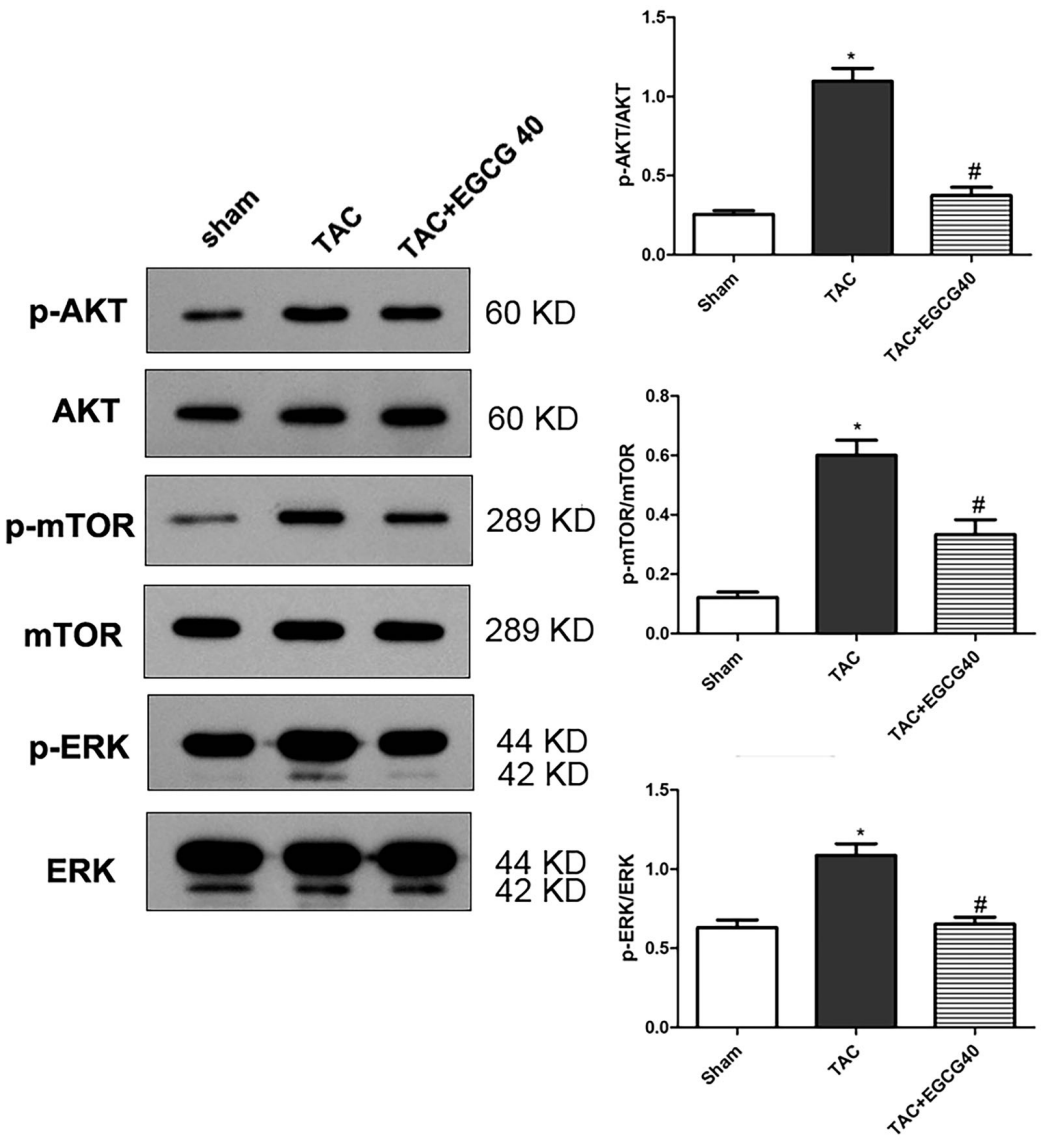
EGCG attenuates the activation of AKT/mTOR signalling and ERK1/2 phosphorylation in mice with TAC. *p < 0.05 as compared with the sham group. ^#^*p* < 0.05 as compared with the TAC group.

To explore the underline mechanism of EGCG treatment for cardiac hypertrophy, we examined the AKT/mTOR signalling pathway and the phosphorylation of ERK. According to the results (Figure 6), phosphorylation of AKT, mTOR, and ERK were significantly increased in response to TAC (*p* < 0.001), approximately two-fold when compared with a sham control group. However, with the administration of EGCG (40 mg/kg), the phosphorylation ratios were dramatically decreased (*p* < 0.01). The result indicated that the signalling was activated following TAC, but inhibited by EGCG treatment.

## Discussion

Myocardial hypertrophy is thought to be an adaptive response to pressure or volume stress and many other problems. Initially, it is adaptively against increased workload or defects in the efficiency of contractile machinery. However, in the longer term, hypertrophy growth may result in the development of heart failure and sudden death (Frey et al. 2004). EGCG is the most abundant catechin extracted from green tea and possessed several biologic activities, such as anti-inflammatory anticancer, antioxidant activities (Liu and Yan 2019). Moreover, a previous study indicated that EGCG can also prevent the risk of cardiovascular disease (Deka and Vita 2011). However, whether the AKT/mTOR signalling pathway was involved in the treatment effect of EGCG for cardiac hypertrophy and fibrosis remains unclear. In this study, we found that EGCG attenuated pressure overload-induced cardiac hypertrophy and the following fibrosis after TAC surgery. More importantly, the activated Akt/mTOR signal pathway during cardiac hypertrophy was inhibited by EGCG treatment.

EGCG was found to be the major polyphenolic compound and exhibit numerous bioactivities. EGCG has also been proved to possess a cardiovascular protective effect, and several signal pathways were implicated in this beneficial process. A study indicated that the treatment of EGCG alleviated cardiac hypertrophy caused by AngII through the inhibition of the NF-kB signal pathway (Cai et al. 2013). Another study showed that the administration of EGCG attenuated cardiac hypertrophy induced by pressure overload, and exhibited through the telomere-dependent apoptotic signal pathway (Sheng et al. 2013). There is another study suggested that ROS-dependent and –independent mechanisms involving inhibition of different intracellular signalling transductional pathways participated in EGCG prevention for the development of cardiac hypertrophy (Li H-L et al. 2006). In this study, EGCG was verified to attenuate the cardiac hypertrophic response induced by TAC pressure overload injury, as evidenced by reduced HW/BW and HW/TL ratios. Furthermore, H&E histological staining also proved the attenuation of cardiac hypertrophic injury. ANP, BNP, and β-MHC are the hypertrophic markers in the heart, the over-expression of these molecules in the ventricular cardiomyocytes are indicating hypertrophy occurrence (Li et al. 2014). The secretions of ANP and BNP from cardiac atria and the ventricles are in response to increased cardiac stretch and functioning to regulate blood pressure (Ellmers et al. 2007). Their expression levels are in parallel with the degree of cardiac dysfunction, therefore, becoming the main indicator for cardiovascular disease (Sergeeva and Christoffels 2013). In our study, the result revealed that EGCG down-regulated the ANP, BNP, and β-MHC protein and gene expression levels, while α-MHC expression level was increased, suggesting that EGCG alleviated cardiac hypertrophy induced by TAC surgery.

Myocardial hypertrophy is often characterised by elevated fibrosis, mostly reactive interstitial fibrosis. The increase of total collagen expression and cross-linking was associated with diastolic dysfunction, and the fibrosis may result in the progression of left ventricular hypertrophy to heart failure (Falcão-Pires et al. 2011). To further verify the cardioprotective effect of EGCG, the myocardial fibrosis situation was also evaluated in this study. Although previous studies have shown that EGCG inhibited cardiac fibrosis in cardiac hypertrophic rats, the underlying mechanism in the EGCG treatment process remains unknown (Sheng et al. 2009). The elevated CTGF expression has been reported in heart failure patient’s heart tissue and the over-expression of CTGF has been demonstrated to be correlated with the severity of fibrosis. A previous study indicated that EGCG reduced collagen synthesis and fibronectin expression induced by abdominal aortic constriction or angiotensin, and in the meantime, alleviated the over-expression of CTGF (Cai et al. 2013). Our results from the present study were following previous studies that collagen I, collagen III, fibronectin, and CTGF gene and protein expressions were all down-regulated by EGCG (Cai et al. 2013). Histological staining results from Masson’s trichrome and Sirius red staining have further confirmed the attenuation of fibrosis.

To determine the molecular mechanisms through which EGCG attenuates cardiac hypertrophy and myocardial fibrosis, we examined the involvement of the AKT/mTOR signalling pathway under pressure overload-induced injury. Our results indicated that EGCG down-regulated the phosphorylation of Akt/mTOR. Therefore, we speculated that Akt/mTOR may be involved in the EGCG treatment process. A previous study showed that activated Akt accelerated the cardiac myocytes growth and increases protein synthesis, and cardiomyocyte-specific Akt over-expression may result in cardiac hypertrophy (Condorelli et al. 2002). Another study also indicated that the cardiac hypertrophy induced by cholesterol was through the activating of the Akt pathway (Lee et al. 2013). The constitutive activation of cardiac Akt suggested that Akt is a vital factor for the increase in myocyte size downstream of phosphoinositide-3-kinase (PI3K) (Shiojima and Walsh 2006). Activated Akt would inhibit TSC1/2 and therefore active mTOR. The administration of mTOR inhibitor rapamycin may decrease the cardiac weight caused by continuous activation of Akt (Shioi et al. 2002). A previous study indicated that EGCG is an ATP-competitive inhibitor of PI3K and mTOR (Van Aller et al. 2011; Chen et al. 2016). In an endometrial cancer model, a pro-drug of EGCG inhibited Akt/mTOR pathway to decrease VEGFA secretion, thus can be used as a promising drug (Wang et al. 2018). In another study performed in human hepatocellular carcinoma, EGCG reduced the levels of p-Akt and phosphorylated extracellular signal-regulated kinase (p-ERK) (Caban et al. 2019). In this present study, EGCG treatment at 40 mg/kg dosages significantly down-regulated the amount of p-AKT to Akt, p-mTOR to mTOR, and p-ERK to ERK, which indicated that the Akt/mTOR signal pathway was involved in the EGCG treatment process for cardiac hypertrophy, and the alleviation of hypertrophy induced fibrosis may also be associated with the inhibition of Akt/mTOR signal pathway. Our finding broadens the understanding for the protective role of EGCG in pressure overload-induced cardiac hypertrophy and provides a promising therapeutic strategy for attenuating the progression of heart failure.

## Conclusions

Our results indicated that the administration of EGCG alleviated the cardiac functions and fibrosis caused by pressure overload. The cardioprotective effect of EGCG may contribute to the inhibition of the Akt/mTOR signal pathway. Our study provided a new promising treatment approach for cardiac hypertrophy. More experiments focusing on upstream or downstream factors of Akt/mTOR are needed.

## References

[CIT0001] Aoyagi T, Matsui T. 2011. Phosphoinositide-3 kinase signaling in cardiac hypertrophy and heart failure. Curr Pharm Des. 17(18):1818–1824. 21631421 10.2174/138161211796390976 PMC3337715

[CIT0005] Caban M, Owczarek K, Chojnacka K, Lewandowska U. 2019. Overview of polyphenols and polyphenol-rich extracts as modulators of IGF-1, IGF-1R, and IGFBP expression in cancer diseases. J Fun Foods. 52:389–407.

[CIT0006] Cai Y, Yu S-S, Chen T-T, Gao S, Geng B, Yu Y, Ye J-T, Liu P-Q. 2013. EGCG inhibits CTGF expression via blocking NF-κB activation in cardiac fibroblast. Phytomedicine. 20(2):106–113. 23141425 10.1016/j.phymed.2012.10.002

[CIT1002] Chen YL, Abraham DJ, Xu SW, Pearson JD, Black CM, Lyons KM, Leask A. 2004. CCN2 (connective tissue growth factor) promotes fibroblast adhesion to fibronectin. Mol Biol Cell. 15(12):5635–5646. 15371538 10.1091/mbc.E04-06-0490 PMC532042

[CIT0007] Chen X, Dong XS, Gao HY, Jiang YF, Jin YL, Chang YY, Chen LY, Wang JH. 2016. Suppression of HSP27 increases the anti-tumor effects of quercetin in human leukemia U937 cells . Mol Med Rep. 13(1):689–696. 26648539 10.3892/mmr.2015.4600 PMC4686121

[CIT1001] Chen J, Wu J, Li L, Zou YZ, Zhu DL, Gao PJ. 2011. Effect of an acute mechanical stimulus on aortic structure in the transverse aortic constriction mouse model. Clin Exp Pharmacol Physiol. 38(9):570–576. 21615773 10.1111/j.1440-1681.2011.05544.x

[CIT1003] Condorelli G, Morisco C, Latronico MV, Claudio PP, Dent P, Tsichlis P, Condorelli G, Frati G, Drusco A, Croce CM, et al. 2002. TNF-alpha signal transduction in rat neonatal cardiac myocytes: definition of pathways generating from the TNF-alpha receptor. Faseb J. 16(13):1732–1737. 12409315 10.1096/fj.02-0419com

[CIT0008] Deka A, Vita JA. 2011. Tea and cardiovascular disease. Pharmacol Res. 64(2):136–145. 21477653 10.1016/j.phrs.2011.03.009 PMC3123419

[CIT1004] Ellmers LJ, Scott NJ, Piuhola J, Maeda N, Smithies O, Frampton CM, Richards AM, Cameron VA. 2007. Npr1-regulated gene pathways contributing to cardiac hypertrophy and fibrosis. J Mol Endocrinol. 38(2):245–257. 17293444 10.1677/jme.1.02138

[CIT0009] Falcão-Pires I, Hamdani N, Borbély A, Gavina C, Schalkwijk CG, van der Velden J, van Heerebeek L, Stienen GJM, Niessen HWM, Leite-Moreira AF, et al. 2011. Diabetes mellitus worsens diastolic left ventricular dysfunction in aortic stenosis through altered myocardial structure and cardiomyocyte stiffness. Circulation. 124(10):1151–1159. 21844073 10.1161/CIRCULATIONAHA.111.025270

[CIT0010] Frey N, Katus HA, Olson EN, Hill JA. 2004. Hypertrophy of the heart: a new therapeutic target? Circulation. 109(13):1580–1589. 15066961 10.1161/01.CIR.0000120390.68287.BB

[CIT0011] Frey N, Olson E. 2003. Cardiac hypertrophy: the good, the bad, and the ugly. Annu Rev Physiol. 65(1):45–79. 12524460 10.1146/annurev.physiol.65.092101.142243

[CIT0013] Heinzel FR, Hohendanner F, Jin G, Sedej S, Edelmann F. 2015. Myocardial hypertrophy and its role in heart failure with preserved ejection fraction. J Appl Physiol (1985)). 119(10):1233–1242. 26183480 10.1152/japplphysiol.00374.2015 PMC4652334

[CIT0014] Higdon JV, Frei B. 2003. Tea catechins and polyphenols: health effects, metabolism, and antioxidant functions. Crit Rev Food Sci Nutr. 43(1):89–143. 12587987 10.1080/10408690390826464

[CIT0015] Ikeda M, Ide T, Fujino T, Matsuo Y, Arai S, Saku K, Kakino T, Oga Y, Nishizaki A, Sunagawa K. 2015. The Akt-mTOR axis is a pivotal regulator of eccentric hypertrophy during volume overload. Sci Rep. 5:15181–15881. 26515499 10.1038/srep15881 PMC4626834

[CIT0018] Lee H, Yoo YS, Lee D, Song EJ. 2013. Cholesterol induces cardiac hypertrophy by activating the AKT pathway. J Steroid Biochem Mol Biol. 138:307–313. 23907017 10.1016/j.jsbmb.2013.07.008

[CIT1005] Li HL, Huang Y, Zhang CN, Liu G, Wei YS, Wang AB, Liu YQ, Hui RT, Wei CM, Williams GM, et al. 2006. Epigallocathechin-3 gallate inhibits cardiac hypertrophy through blocking reactive oxidative species-dependent and -independent signal pathways. Free Radic Biol Med. 40(10):1756–1775. 16767845 10.1016/j.freeradbiomed.2006.01.005

[CIT0019] Li C, Li X, Gao X, Zhang R, Zhang Y, Liang H, Xu C, Du W, Zhang Y, Liu X, et al. 2014. MicroRNA-328 as a regulator of cardiac hypertrophy. Int J Cardiol. 173(2):268–276. 24631114 10.1016/j.ijcard.2014.02.035

[CIT0020] Lipson Kenneth E, Carol W, Yuchin T, Spong S. 2012. CTGF is a central mediator of tissue remodeling and fibrosis and its inhibition can reverse the process of fibrosis. Fibrogenesis Tissue Repair. 5(S1):24–43. 10.1186/1755-1536-5-S1-S24 PMC3368796 23259531

[CIT0021] Liu B, Yan W. 2019. Lipophilization of EGCG and effects on antioxidant activities. Food Chem. 272:663–669. 30309596 10.1016/j.foodchem.2018.08.086

[CIT0024] Liu Z-W, Zhu H-T, Chen K-L, Dong X, Wei J, Qiu C, Xue J-H. 2013. Protein kinase RNA-like endoplasmic reticulum kinase (PERK) signaling pathway plays a major role in reactive oxygen species (ROS)-mediated endoplasmic reticulum stress-induced apoptosis in diabetic cardiomyopathy. Cardiovasc Diabetol. 12:158–166. 24180212 10.1186/1475-2840-12-158 PMC4176998

[CIT0025] Molkentin JD, Dorn II GW. 2001. Cytoplasmic signaling pathways that regulate cardiac hypertrophy. Annu Rev Physiol. 63(1):391–426. 11181961 10.1146/annurev.physiol.63.1.391

[CIT0026] Negri A, Naponelli V, Rizzi F, Bettuzzi S. 2018. Molecular targets of epigallocatechin-gallate (EGCG): a special focus on signal transduction and cancer. Nutrients. 10:1936–1952. 10.3390/nu10121936 PMC6315581 30563268

[CIT0028] Sergeeva IA, Christoffels VM. 2013. Regulation of expression of atrial and brain natriuretic peptide, biomarkers for heart development and disease. Biochim Biophys Acta - Molecular Basis of Disease. 1832(12):2403–2413. 10.1016/j.bbadis.2013.07.003 23851052

[CIT0029] Sheng R, Gu Z-L, Xie M-L. 2013. Epigallocatechin gallate, the major component of polyphenols in green tea, inhibits telomere attrition mediated cardiomyocyte apoptosis in cardiac hypertrophy. Int J Cardiol. 162(3):199–209. 22000973 10.1016/j.ijcard.2011.07.083

[CIT0030] Sheng R, Gu Z-l, Xie M-l, Zhou W-X, Guo C-Y. 2009. EGCG inhibits proliferation of cardiac fibroblasts in rats with cardiac hypertrophy. Planta Med. 75(2):113–120. 19096994 10.1055/s-0028-1088387

[CIT0031] Sheng R, Gu Z, Xie M, Zhou W, Guo C. 2006. EGCG inhibition against collagen accumulation and cell proliferation in cardiac hypertrophy. Chinese Pharmacol Bull. 22:1095–2009.

[CIT0032] Sheng R, Gu Z, Xie M, Zhou W, Guo C. 2007. EGCG inhibits cardiomyocyte apoptosis in pressure overload-induced cardiac hypertrophy and protects cardiomyocytes from oxidative stress in rats 1. Acta Pharmacol Sin. 28(2):191–201. 17241521 10.1111/j.1745-7254.2007.00495.x

[CIT0033] Shioi T, McMullen JR, Kang PM, Douglas PS, Obata T, Franke TF, Cantley LC, Izumo S. 2002. Akt/protein kinase B promotes organ growth in transgenic mice. Mol Cell Biol. 22(8):2799–2809. 11909972 10.1128/MCB.22.8.2799-2809.2002 PMC133704

[CIT1007] Shiojima I, Walsh K. 2006. Regulation of cardiac growth and coronary angiogenesis by the Akt/PKB signaling pathway. Genes Dev. 20(24):3347–3365. 17182864 10.1101/gad.1492806

[CIT0034] Sugden PH. 1999. Signaling in myocardial hypertrophy: life after calcineurin? Circ Res. 84(6):633–646. 10189351 10.1161/01.res.84.6.633

[CIT1008] Tagashira H, Bhuiyan S, Shioda N, Hasegawa H, Kanai H, Fukunaga K. 2010. Sigma1-receptor stimulation with fluvoxamine ameliorates transverse aortic constriction-induced myocardial hypertrophy and dysfunction in mice. Am J Physiol Heart Circ Physiol. 299(5):H1535–H1545. 20802134 10.1152/ajpheart.00198.2010

[CIT0035] Van Aller GS, Carson JD, Tang W, Peng H, Zhao L, Copeland RA, Tummino PJ, Luo L. 2011. Epigallocatechin gallate (EGCG), a major component of green tea, is a dual phosphoinositide-3-kinase/mTOR inhibitor. Biochem Biophys Res Commun. 406(2):194–199. 21300025 10.1016/j.bbrc.2011.02.010

[CIT0037] Wang J, Man GCW, Chan TH, Kwong J, Wang CC. 2018. A prodrug of green tea polyphenol (-)-epigallocatechin-3-gallate (Pro-EGCG) serves as a novel angiogenesis inhibitor in endometrial cancer. Cancer Lett. 412:10–20. 29024813 10.1016/j.canlet.2017.09.054

[CIT1009] Weber KT, Sun Y, Tyagi SC, Cleutjens JP. 1994. Collagen network of the myocardium: function, structural remodeling and regulatory mechanisms. J Mol Cell Cardiol. 26(3):279–292. 8028011 10.1006/jmcc.1994.1036

[CIT0039] Yan H-J, Qi G-Q, Ma Y. 2019. Effect of propofol on myocardial ischemia-reperfusion injury through MAPK/ERK pathway. Eur Rev Med Pharmacol Sci. 23:11051–11061. 31858577 10.26355/eurrev_201912_19813

[CIT0038] Yang E, Lee J, Lee S, Kim E, Moon Y, Jung Y, Park S, Cho M. 2014. EGCG attenuates autoimmune arthritis by inhibition of STAT3 and HIF-1α with Th17/Treg control. PloS One. 9(2):86–102. 10.1371/journal.pone.0086062 PMC3928092 24558360

